# Association Between Varicella Vaccination Status and Self-Reported Contact History Among Confirmed Varicella Cases in Chaoyang District, Beijing, 2017–2025

**DOI:** 10.3390/vaccines14070617

**Published:** 2026-07-14

**Authors:** Yang Cao, Hao Wang, Ziwei Zhao, Zhen Li, Hai Fang

**Affiliations:** 1 Chaoyang District Center for Disease Control and Prevention, Beijing 100021, China; 2 School of Health Humanities, Peking University, Beijing 100191, China; 3 China Center for Health Development Studies, Peking University, Beijing 100191, China; 4 Key Laboratory of Health System Reform and Governance of National Health Commission, Peking University, Beijing 100191, China

**Keywords:** varicella, breakthrough infection, vaccination, contact tracing, epidemiological surveillance, herpes zoster

## Abstract

**Objectives:** To investigate associations between varicella vaccination and self-reported contact history with varicella or herpes zoster among clinically diagnosed varicella cases in Chaoyang District, Beijing, China. **Methods:** A retrospective observational study was conducted among 4441 clinically diagnosed varicella cases reported from 2017 to 2025. Multivariable logistic regression examined associations between vaccination status, number of doses, time since vaccination, and reported contact history with varicella and/or herpes zoster, adjusting for age, sex, and year of diagnosis. **Results:** Vaccinated cases had significantly higher odds of reporting contact with varicella or zoster compared with unvaccinated cases (OR = 1.34, 95% CI: 1.14–1.58). Both 1-dose and 2-dose recipients showed similar associations. A gradient of increasing odds was observed with longer time since vaccination, reaching OR = 3.17 (95% CI: 1.39–7.22) for 10 or more years since last dose. Associations were primarily driven by reported varicella contact rather than herpes zoster contact. Sensitivity analysis confirmed robustness of findings. **Conclusions:** Varicella vaccination was positively associated with reporting an identifiable exposure source. Vaccination status may influence completeness of exposure ascertainment in varicella surveillance and should be considered when interpreting contact tracing data.

## 1. Introduction

Varicella (chickenpox) is a highly contagious disease caused by varicella-zoster virus (VZV), transmitted primarily via respiratory droplets and direct contact with vesicular lesions [1]. After primary infection, VZV establishes lifelong latency in sensory ganglia and may reactivate as herpes zoster (shingles). Individuals with herpes zoster can transmit VZV to susceptible contacts, leading to primary varicella infection [2]. Thus, both active varicella cases and herpes zoster cases may serve as sources of VZV transmission in the community.

The live attenuated varicella vaccine, first licensed in the 1990s, has been a cornerstone of varicella prevention [3]. A global meta-analysis estimated that a single dose of varicella vaccine provides approximately 81% effectiveness against varicella of any severity [4], while two-dose schedules have demonstrated effectiveness exceeding 95% [5]. Routine vaccination programs have achieved substantial reductions in varicella incidence, hospitalizations, and mortality in countries with high coverage [6]. The World Health Organization has recommended that countries with sufficient resources and the capacity to sustain high coverage consider incorporating varicella vaccine into routine childhood immunization schedules [7].

Despite proven effectiveness, breakthrough varicella—defined as varicella occurring more than 42 days after vaccination—continues to be observed, particularly in settings where only a single dose is routinely administered [8,9]. Breakthrough cases are generally milder than varicella in unvaccinated individuals, presenting with fewer lesions and lower complication rates [10]. Nevertheless, vaccinated individuals with breakthrough disease retain the capacity to transmit VZV and have been implicated in outbreaks in schools and childcare facilities, even within highly vaccinated populations [11,12]. Multiple longitudinal studies have demonstrated that vaccine-induced immunity wanes over time, with the risk of breakthrough infection increasing progressively with longer intervals since vaccination [13,14,15]. This evidence of waning immunity has been a major factor supporting the adoption of two-dose vaccination schedules in many countries.

In China, varicella vaccine has been available since the early 2000s, initially as a self-paid vaccine not included in the National Immunization Program (NIP) [7]. Several municipalities have since incorporated varicella vaccination into their local immunization programs. Despite increasing coverage, varicella continues to be one of the most commonly reported vaccine-preventable infectious diseases in China [16], underscoring the need for continued epidemiological investigation to inform prevention and control strategies.

Routine surveillance of varicella includes epidemiological investigation of individual cases, a key component of which is the assessment of pre-onset exposure history—specifically, whether the patient had known contact with a varicella or herpes zoster case during the incubation period. This self-reported contact history provides essential information for identifying transmission chains and evaluating the effectiveness of containment measures. However, the completeness and accuracy of self-reported exposure data may be influenced by a variety of factors, including the respondent’s health awareness, recall ability, and engagement with the healthcare system. Research on COVID-19 contact tracing has demonstrated that the ability to identify a transmission source varies substantially depending on the proximity and setting of exposure, with household and institutional contacts being far more readily ascertained than community-level exposures [17]. Furthermore, studies of vaccine effectiveness have increasingly recognized the “healthy vaccinee effect,” whereby vaccinated individuals systematically differ from unvaccinated individuals not only in immune status but also in health-related behaviors, health literacy, and engagement with healthcare systems [18]. These behavioral correlates of vaccination may extend to the recognition and reporting of disease-related exposures. In the varicella-specific context, breakthrough cases have been shown to cluster disproportionately within identifiable outbreaks in institutional settings [19], where exposure sources are more readily ascertained by both cases and public health investigators. Despite these converging lines of evidence, whether vaccination status is systematically associated with the likelihood of reporting a known exposure source among confirmed cases of vaccine-preventable diseases has not been directly examined.

The present study aimed to investigate the associations between varicella vaccination characteristics, including vaccination status, number of doses received, and time since vaccination, and self-reported contact history with varicella or herpes zoster among clinically diagnosed varicella cases in Chaoyang District, Beijing, from 2017 to 2025. We further explored whether any observed associations varied by type of reported contact (varicella versus herpes zoster) and assessed the robustness of findings through sensitivity analyses addressing missing vaccination data. These analyses are intended to improve interpretation of exposure information recorded in surveillance data and to provide insights relevant to contact tracing and outbreak investigation. Importantly, the study does not aim to infer causal effects of vaccination on contact reporting, but rather to describe patterns of exposure ascertainment within routine surveillance settings.

## 2. Methods

### 2.1. Study Design and Population

This is a retrospective observational study conducted among clinically diagnosed varicella cases reported in Chaoyang District, Beijing, China, between 2017 and 2025. Varicella cases were identified through the routine notifiable disease surveillance system and diagnosed by clinicians according to standardized clinical diagnostic criteria used in local public health practice. Laboratory confirmation was not routinely required for case reporting in this surveillance system.

In 2025, Chaoyang District had a population of approximately 3.419 million [20]. Individual-level epidemiological investigation data were extracted from routine surveillance records, which were collected through structured field investigations conducted by trained public health professionals following case notification. These records included demographic characteristics, varicella vaccination history, pre-onset exposure history, and diagnostic information.

Contact history was ascertained during routine epidemiological investigations and recorded in the surveillance system based on information reported by cases or their caregivers, as well as exposures identified during outbreak or institutional investigations when applicable. Both self-reported and investigator-identified exposure sources were documented within the same standardized surveillance framework.

A total of 4441 clinically diagnosed varicella cases were included in the study. Cases with missing vaccination status were excluded from the primary analysis but retained for sensitivity analysis, yielding an effective analytic sample of 3696 cases for the primary analysis. For analyses involving time since vaccination, a consistent subsample of 2470 cases with complete and valid first-dose and last-dose interval data was used.

### 2.2. Variable Definitions

All individuals included in this study were clinically diagnosed with varicella. Contact history was ascertained through routine epidemiological investigations conducted after case notification in the notifiable disease surveillance system. The ascertainment process followed a standardized workflow implemented by trained public health professionals. For cases identified as part of institutional or outbreak settings (e.g., schools, childcare centers, or other clustered settings), exposure information was obtained from outbreak investigation reports and institutional surveillance records, which included identified links between cases within the same setting. For sporadic community cases, contact history was obtained primarily through structured interviews with cases or their caregivers during field investigations. Information on potential exposure to varicella or herpes zoster cases within the incubation period was recorded based on respondent reports. All contact information was entered into the standardized surveillance database. Both investigator-identified exposures (e.g., outbreak-linked contacts) and self-reported exposures (e.g., household or community contacts reported by caregivers) were recorded within the same data system and used to construct the analytic exposure variables. Three binary outcome variables (yes vs. no) were constructed: the primary outcome was reported contact with varicella or zoster (any exposure to either varicella or herpes zoster cases), and two secondary outcomes were reported contact with varicella alone and reported contact with zoster alone.

Four exposure variables related to varicella vaccination were examined. The first was varicella vaccination status, classified as vaccinated versus unvaccinated. The second was the number of varicella vaccine doses received, categorized as 0 doses (unvaccinated), 1 dose, and 2 doses; cases who received three or more doses were reclassified as 2 doses, and vaccinated individuals with unreported dose counts were assigned to the 1-dose category. The third and fourth exposure variables were the time intervals from the first and last varicella vaccine dose to disease onset, respectively. These intervals were categorized into five groups: 43 days to less than 1 year (reference), 1 to less than 3 years, 3 to less than 5 years, 5 to less than 10 years, and 10 years or more. Cases in which disease onset occurred within 42 days after vaccination were excluded from the time-interval analyses. This exclusion was based on the consideration that varicella vaccine requires approximately 4 to 6 weeks to induce adequate seroconversion and confer immune protection [3,7]. Varicella cases developing within this window are unlikely to reflect vaccine failure or waning immunity, as the vaccine has not yet had sufficient time to elicit a protective immune response. Therefore, the lower bound of the time-since-vaccination interval was set at 43 days (i.e., the first day beyond the 42-day seroconversion window), and the reference category was defined as 43 days to less than 1 year.

Three covariates were included in all adjusted models: age in years (continuous), sex (male vs. female), and year of diagnosis (categorical, with 2017 as the reference group). These variables were selected a priori based on their established or plausible associations with both vaccination history and exposure reporting and were consistently available in the surveillance dataset. The regression modeling strategy was guided by data availability within the routine surveillance system and prior knowledge of potential confounders.

As the data were derived from routine infectious disease surveillance, information on several potential confounders was not systematically collected, including contextual factors such as school or childcare attendance, household socioeconomic status, and caregiver education, as well as clinical factors such as underlying immunosuppression, medication use (e.g., corticosteroids), and other behavioral or psychosocial characteristics. Therefore, these variables could not be incorporated into the adjusted analyses, and residual confounding due to unmeasured factors cannot be excluded.

### 2.3. Statistical Analysis

Baseline characteristics of study participants were stratified according to whether they reported a confirmed contact history with varicella or zoster. Differences in varicella vaccination status were assessed by confirmed contact with varicella and/or zoster cases. Continuous variables were presented as means with standard deviations and compared using Student’s *t*-test. Categorical variables were presented as frequencies with column percentages and compared using Pearson’s chi-square test.

Multivariable logistic regression models were used to estimate odds ratios (ORs) and 95% confidence intervals (CIs) for the associations between vaccination-related exposure and confirmed contacts with varicella and/or zoster cases. All models were adjusted for age, sex, and year of diagnosis. Four sets of models were constructed. The first set examined the association between vaccination status (vaccinated vs. unvaccinated) and confirmed contacts of varicella and/or zoster cases. The second set examined the association between number of vaccine doses (0, 1, or 2 doses) and confirmed contacts, with 0 doses as the reference group. The third and fourth sets examined the associations between time since first vaccine dose and time since last vaccine dose, respectively, and confirmed contacts, with the 43 days to less than 1 year interval as the reference group. The time-interval analyses were restricted to the consistent subsample of 2470 vaccinated cases with available varicella vaccination date information.

To assess the robustness of the primary findings to potential bias introduced by missing vaccination data, a sensitivity analysis was performed in which all cases with unreported vaccination status were reclassified as unvaccinated. The multivariable logistic regression models for vaccination status and number of doses were then re-estimated using the full sample of 4441 cases, with the same set of covariates. Results were compared with those from the primary analysis to evaluate consistency.

All data management and statistical analyses were performed using Stata SE 19. All statistical tests were two-sided, and a *p*-value of less than 0.05 was considered statistically significant.

## 3. Results

### 3.1. Descriptive Characteristics of Study Participants

A total of 4441 clinically diagnosed varicella cases were included in the study, of whom 1545 (34.8%) reported contact with varicella or zoster cases within three weeks prior to disease onset and 2896 (65.2%) did not. The mean age of participants was 14.2 years (SD = 7.5), with no significant difference between those with and without reported contact history (14.0 vs. 14.3 years, *p* = 0.204). Males accounted for 55.3% of the total sample, and the proportion of males did not differ significantly between the two groups (57.0% vs. 54.4%, *p* = 0.105).

Among the 3696 cases with known vaccination status, 2724 (73.7%) had received at least one dose of varicella vaccine. The proportion of vaccinated individuals was significantly higher among those who reported a contact history than among those who did not (77.2% vs. 71.7%, *p* < 0.001). With respect to the number of vaccine doses, 48.4% of cases had received 1 dose and 25.3% had received 2 doses, and the distribution of dose categories differed significantly between the two groups (*p* = 0.001). Among the 2470 vaccinated cases with available vaccination date information, the distribution of time since first varicella dose did not differ significantly between those with and without reported contact history (*p* = 0.233), nor did the distribution of time since last varicella dose (*p* = 0.106). In both interval variables, the majority of cases fell into the 5–10 years and ≥10 years categories, together accounting for over 70% of the subsample (Table 1).

### 3.2. Association Between Vaccination Status and Reported Contact History

In multivariable logistic regression models adjusted for age, sex, and year of diagnosis, vaccinated cases had significantly higher odds of reporting contact with varicella or zoster compared with unvaccinated cases (OR = 1.34, 95% CI: 1.14–1.58). When the outcome was restricted to reported contact with varicella alone, the association remained significant and was of similar magnitude (OR = 1.36, 95% CI: 1.12–1.64). For reported contact with zoster alone, the point estimate was in the same direction but did not reach statistical significance (OR = 1.18, 95% CI: 0.93–1.51). Among the covariates, age was positively associated with reported contact with varicella (OR = 1.02 per year, 95% CI: 1.01–1.04) but inversely associated with reported contact with zoster (OR = 0.97 per year, 95% CI: 0.96–0.99). Sex was not significantly associated with any of the three outcomes. Year of diagnosis showed notable variation across outcomes, with particularly elevated odds of reporting zoster contact observed in 2020 (OR = 4.31, 95% CI: 2.85–6.51) and 2022 (OR = 6.12, 95% CI: 3.94–9.51) relative to 2017 (Table 2).

### 3.3. Association Between Number of Varicella Doses and Reported Contact History

When vaccination was modeled as number of doses, both 1-dose and 2-dose recipients showed significantly higher odds of reporting contact with varicella or zoster compared with unvaccinated cases (1 dose: OR = 1.34, 95% CI: 1.13–1.60; 2 doses: OR = 1.35, 95% CI: 1.10–1.65). Similar patterns were observed for reported contact with varicella alone (1 dose: OR = 1.36, 95% CI: 1.12–1.66; 2 doses: OR = 1.35, 95% CI: 1.06–1.71). For reported contact with zoster alone, neither 1-dose nor 2-dose vaccination was significantly associated with the outcome (1 dose: OR = 1.17, 95% CI: 0.91–1.52; 2 doses: OR = 1.20, 95% CI: 0.90–1.60). Notably, the odds ratios for 1-dose and 2-dose recipients were nearly identical across all three outcomes, suggesting no incremental effect of the second dose on the likelihood of reporting a contact history (Table 3).

### 3.4. Association Between Time Since First Varicella Dose and Reported Contact History

Among the 2470 vaccinated cases with available vaccination date information, a trend of increasing odds of reporting contact with varicella or zoster was observed with longer time since the first vaccine dose, relative to the reference group of 43 days to less than 1 year. The associations were not statistically significant for the 1–3-year (OR = 2.21, 95% CI: 0.86–5.65) and 3–5-year intervals (OR = 2.43, 95% CI: 0.95–6.24), but reached significance for the 5–10-year interval (OR = 2.91, 95% CI: 1.15–7.40) and the ≥10-year interval (OR = 3.17, 95% CI: 1.20–8.41). When the outcome was restricted to reported contact with varicella alone, the point estimates followed a similar ascending pattern (ranging from OR = 1.50 for 1–3 years to OR = 2.81 for ≥10 years), although none of the individual interval categories reached statistical significance. For reported contact with zoster alone, no significant associations were observed across any interval category (Table 4).

### 3.5. Association Between Time Since Last Varicella Dose and Reported Contact History

A similar gradient was observed for time since the last vaccine dose. For reported contact with varicella or zoster, the odds ratios increased progressively from 2.11 (95% CI: 0.97–4.59) for the 1–3-year interval to 3.17 (95% CI: 1.39–7.22) for the ≥10-year interval, with the associations reaching statistical significance for the 3–5-year (OR = 2.38, 95% CI: 1.09–5.18), 5–10-year (OR = 2.94, 95% CI: 1.36–6.36), and ≥10-year intervals. For reported contact with varicella alone, a pronounced ascending trend was also evident, with the 5–10-year interval (OR = 3.19, 95% CI: 1.11–9.22) and the ≥10-year interval (OR = 4.15, 95% CI: 1.37–12.54) reaching statistical significance. In contrast, no significant associations were found between time since last dose and reported contact with zoster for any interval category (Table 5).

### 3.6. Sensitivity Analysis

In the sensitivity analysis in which all 745 cases with unreported vaccination status were reclassified as unvaccinated, the full sample of 4441 cases was analyzed. Vaccinated cases remained significantly more likely to report contact with varicella compared with the expanded unvaccinated group (OR = 1.52, 95% CI: 1.28–1.79). The dose-specific estimates were also consistent, with odds ratios of 1.53 (95% CI: 1.28–1.82) for 1 dose and 1.49 (95% CI: 1.20–1.85) for 2 doses relative to 0 doses. These estimates were slightly larger in magnitude than those observed in the primary analysis, likely reflecting the dilution of the unvaccinated reference group with potentially misclassified cases. Overall, the sensitivity analysis confirmed the robustness of the primary findings, indicating that the observed associations were not materially affected by missing vaccination status data (Supplementary Table S1).

## 4. Discussion

In this retrospective study of 4441 clinically diagnosed varicella cases in Chaoyang District, Beijing, we found that varicella vaccination was significantly and positively associated with self-reported contact history with varicella or herpes zoster prior to disease onset. This association was observed consistently among both 1-dose and 2-dose recipients was primarily driven by reported contact with varicella rather than herpes zoster and showed an increasing trend with longer time since vaccination. The findings remained robust in sensitivity analysis in which all cases with unreported vaccination status were reclassified as unvaccinated.

The observation that vaccinated varicella cases were more likely to report an identifiable exposure source may be explained by several interrelated mechanisms. First, from a biological perspective, vaccine-induced immunity—even when insufficient to prevent infection entirely—raises the threshold of viral inoculum required to establish productive infection [4,13]. Breakthrough varicella may therefore be more likely to result from much closer, sustained contact with an infectious individual, such as a household member or classmate, rather than from brief or casual community exposure [11]. Such close contacts are inherently more recognizable and memorable, which may increase the likelihood that vaccinated cases identify and report a specific exposure source of varicella and/or herpes zoster during epidemiological investigation.

Second, vaccination status may serve as a proxy for broader health-related awareness and health-seeking behavior. Families who choose to vaccinate their children tend to be more health-literate [21] and more engaged with preventive healthcare services [22]. These behavioral characteristics could translate into greater awareness of varicella or herpes zoster cases in their social environment and a higher likelihood of recognizing and recalling relevant exposures. While this behavioral explanation cannot be directly verified with the available surveillance data, it represents a plausible and complementary pathway underlying the observed association.

Third, the epidemiological context in which breakthrough varicella occurs may itself facilitate exposure identification. Breakthrough cases frequently arise during recognized outbreaks in institutional settings such as schools and childcare centers [8], where an index case has often already been identified by public health authorities and parents or staff have been alerted. In such settings, secondary cases are more likely to be aware of their source of exposure compared with cases arising from unrecognized community transmission.

The observed gradient of increasing odds of reporting contact history with longer time since vaccination is particularly noteworthy. For the composite outcome of varicella or zoster contact, the odds ratio increased from approximately 2.2 for 1–3 years post-vaccination to 3.2 for 10 or more years since the first dose, with a similar pattern observed for time since the last dose. This temporal trend is broadly consistent with the well-documented waning of vaccine-induced immunity over time [23]. As vaccine-derived protection diminishes, breakthrough infection may increasingly occur in recognizable epidemiological contexts—such as household or school exposures—that facilitate source identification. However, several methodological considerations temper the interpretation of these findings. The reference category (43 days to less than 1-year post-vaccination) included only a small number of cases (*N* = 29 for first dose; *N* = 43 for last dose), resulting in wide confidence intervals and limited statistical precision. Furthermore, cases developing varicella shortly after vaccination may disproportionately represent primary vaccine failure—in which the vaccine fails to induce seroconversion [24]—and such individuals may be immunologically similar to unvaccinated persons, potentially acquiring infection through lower-intensity exposures that are harder to identify. Age-related confounding, although adjusted for in the models, cannot be entirely excluded, as individuals vaccinated longer ago tend to be older and may differ in exposure patterns and reporting behaviors.

The finding that 1-dose and 2-dose recipients showed virtually identical odds of reporting contact history (OR approximately 1.34–1.36 for the composite outcome) merits discussion. One possible explanation is a threshold biological effect: a single dose of varicella vaccine may already sufficiently raise the level of exposure intensity required for breakthrough infection, such that most breakthrough cases, regardless of the number of doses received, arise from close, sustained contacts that are inherently more recognizable. Among those who develop breakthrough varicella despite two-dose vaccination, the epidemiological context of infection acquisition may be similar to that of one-dose breakthrough cases, as both groups have, by definition, experienced exposures of sufficient intensity to overcome vaccine-induced immunity. Additionally, two-dose breakthrough cases may represent a subpopulation with suboptimal immune responses, whose susceptibility characteristics resemble those of one-dose recipients who experience vaccine failure. While behavioral and contextual correlates of vaccination cannot be excluded as contributing factors, the observed pattern is also consistent with a biological threshold mechanism that plateaus after the first dose among individuals who ultimately develop breakthrough disease.

A noteworthy finding from the temporal covariate analysis was the pronounced shift in reported contact patterns during and after 2020. Relative to 2017, the odds of reporting herpes zoster contact were substantially elevated from 2020 onward (ranging from OR = 2.10 in 2023 to OR = 6.12 in 2022), whereas the odds of reporting varicella contact declined notably from 2022 (OR = 0.33 in 2022; OR = 0.58 in 2025). This reciprocal pattern may partly reflect changes in varicella circulation associated with non-pharmaceutical interventions during the COVID-19 pandemic [25], as well as broader secular trends in VZV epidemiology. With reduced varicella transmission in the community, herpes zoster—resulting from endogenous reactivation of latent VZV rather than person-to-person transmission—may have become a relatively more prominent recognized source of varicella infection. This observation highlights the dual role of varicella and herpes zoster as complementary sources of VZV transmission and suggests that shifts in population-level transmission dynamics can alter the composition of reported exposure sources.

The consistently stronger association between vaccination and reported varicella contact compared with reported zoster contact across all models likely reflects the differing recognizability of these two exposure types. Varicella is a readily identifiable childhood illness that often occurs in visible clusters within schools and households, making the source of transmission apparent to both cases and investigators [1]. In contrast, herpes zoster typically occurs sporadically among older adults, and its role as a potential source of varicella may not be recognized by the exposed individual or their family [2]. The non-significant association between vaccination and reported zoster contact (OR = 1.18, 95% CI: 0.93–1.51) may thus reflect the inherently lower recognizability of zoster as a transmission source rather than a genuinely absent association.

Several limitations should be considered when interpreting the findings. First, the retrospective, cross-sectional design precludes causal inference, and the direction of association between vaccination and exposure reporting cannot be definitively established. Second, for sporadic community cases, contact history relied on patient or caregiver self-report and may be subject to recall bias, although cases linked to institutional outbreaks had contact sources identified through public health investigation records [26]. Importantly, the bias is likely differential: vaccinated individuals and their families may be more attentive to disease-related contacts, which could inflate the observed association. Third, the study cannot distinguish between genuinely differential exposure (vaccinated cases truly having more identifiable contacts) and differential reporting (vaccinated cases being more likely to recall and report contacts). These two mechanisms are not mutually exclusive and may act simultaneously. Fourth, all cases were clinically diagnosed without laboratory confirmation, introducing the possibility of diagnostic misclassification. However, the clinical diagnosis of varicella is generally considered highly accurate owing to its distinctive vesicular rash presenting in successive crops, and previous studies have demonstrated a high positive predictive value of clinical varicella diagnosis in both outbreak and non-outbreak settings [27]. This reflects standard practice for varicella surveillance in China. Fifth, the covariates available for adjustment were limited to age, sex, and year of diagnosis. As a result, potentially important confounders, such as socioeconomic status, educational level, household size, school or childcare attendance, and occupation, were not available in the surveillance dataset. Therefore, residual confounding due to unmeasured factors cannot be excluded, and the adjusted estimates should be interpreted with caution. Finally, vaccination data were missing for 745 cases (16.8%); however, the sensitivity analysis reclassifying these cases as unvaccinated produced consistent and slightly larger effect estimates, supporting the robustness of the primary findings.

## 5. Conclusions

In conclusion, this study identified an association between varicella vaccination and self-reported contact history among clinically diagnosed varicella cases, with a stronger association observed with longer time since vaccination. These findings suggest differences in exposure ascertainment within varicella surveillance data by vaccination status, which may reflect variations in recall, reporting behavior, or case investigation processes, rather than a causal effect of vaccination on contact reporting. The results should therefore be interpreted in the context of surveillance-based data collection and contact tracing practices. The findings also highlight the evolving role of herpes zoster as a reported source of varicella infection, particularly during periods of reduced varicella circulation. Future studies incorporating objective exposure ascertainment methods, such as molecular genotyping to link cases or prospective contact diaries, would be valuable for elucidating the relative contributions of biological susceptibility, exposure patterns, and reporting processes to the observed associations.

## Figures and Tables

**Table 1:** Varicella Vaccination Status and Demographic Characteristics of Study Participants.

Characteristic	Total (*N* = 4441)	With Reported Contact History (*N* = 1545)	Without Reported Contact History (*N* = 2896)	*p*-Value
**Varicella Vaccination Status**	(*N* = 3696)	(*N* = 1347)	(*N* = 2349)	<0.001
**Unvaccinated**	972 (26.3)	307 (22.8)	665 (28.3)	
**Vaccinated**	2724 (73.7)	1040 (77.2)	1684 (71.7)	
**Number of Varicella Doses**	(*N* = 3696)	(*N* = 1347)	(*N* = 2349)	0.001
**0 doses**	972 (26.3)	307 (22.8)	665 (28.3)	
**1 dose**	1789 (48.4)	682 (50.6)	1107 (47.1)	
**2 doses**	935 (25.3)	358 (26.6)	577 (24.6)	
**Time Since First Varicella Dose**	(*N* = 2470)	(*N* = 941)	(*N* = 1529)	0.233
**43 days–1 year**	29 (1.2)	6 (0.6)	23 (1.5)	
**1–3 years**	282 (11.4)	100 (10.6)	182 (11.9)	
**3–5 years**	267 (10.8)	97 (10.3)	170 (11.1)	
**5–10 years**	834 (33.8)	326 (34.6)	508 (33.2)	
**≥10 years**	1058 (42.8)	412 (43.8)	646 (42.2)	
**Time Since Last Varicella Dose**	(*N* = 2470)	(*N* = 941)	(*N* = 1529)	0.106
**43 days–1 year**	43 (1.7)	9 (1.0)	34 (2.2)	
**1–3 years**	308 (12.5)	109 (11.6)	199 (13.0)	
**3–5 years**	311 (12.6)	114 (12.1)	197 (12.9)	
**5–10 years**	868 (35.1)	343 (36.5)	525 (34.3)	
**≥10 years**	940 (38.1)	366 (38.9)	574 (37.5)	
**Age, years**				0.204
**Mean (SD)**	14.2 (7.5)	14.0 (7.3)	14.3 (7.6)	
**Sex, male**				0.105
**n (%)**	2456 (55.3)	880 (57.0)	1576 (54.4)	

*Note.* Values are presented as mean (standard deviation, SD) for continuous variables, or n (column percentage) for categorical variables. Reported contact history was defined as self-reported exposure to varicella or herpes zoster cases within 3 weeks prior to disease onset. *p*-values were calculated using Student’s *t*-test for continuous variables and Pearson’s Chi-square test for categorical variables. Missing data exist for vaccination status, number of doses, and time since vaccine dose, leading to smaller sample sizes for these variables (total *N* = 3696 for vaccination status and dose count; total *N* = 2470 for time since first/last dose).

**Table 2:** Multivariable Logistic Regression for Vaccination Status and Reported Contact History.

Characteristic	Reported Contact with Varicella or Zoster OR (95% CI)	Reported Contact with Varicella OR (95% CI)	Reported Contact with Zoster OR (95% CI)
**Vaccinated (vs. Unvaccinated)**	1.34 *** (1.14, 1.58)	1.36 ** (1.12, 1.64)	1.18 (0.93, 1.51)
**Sex, male (vs. female)**	1.04 (0.90, 1.19)	1.15 (0.98, 1.35)	0.86 (0.70, 1.04)
**Age, per 1-year increase**	1.01 (0.99, 1.02)	1.02 *** (1.01, 1.04)	0.97 *** (0.96, 0.99)
**Year of diagnosis (vs. 2017)**			
**2018**	1.10 (0.87, 1.39)	1.02 (0.79, 1.31)	1.27 (0.84, 1.92)
**2019**	1.10 (0.87, 1.40)	1.01 (0.79, 1.30)	1.29 (0.85, 1.95)
**2020**	2.15 *** (1.62, 2.86)	1.07 (0.79, 1.47)	4.31 *** (2.85, 6.51)
**2021**	1.36 * (1.03, 1.80)	0.80 (0.58, 1.10)	3.01 *** (1.97, 4.59)
**2022**	1.39 (0.99, 1.94)	0.33 *** (0.20, 0.55)	6.12 *** (3.94, 9.51)
**2023**	0.93 (0.70, 1.25)	0.63 ** (0.45, 0.88)	2.10 ** (1.33, 3.33)
**2024**	1.22 (0.92, 1.61)	0.72 * (0.53, 0.99)	2.86 *** (1.87, 4.37)
**2025**	1.09 (0.82, 1.45)	0.58 ** (0.41, 0.82)	2.99 *** (1.95, 4.59)
**Observations (N)**	3696	3696	3696

*Note.* OR = Odds Ratio; CI = Confidence Interval. Reference group for the primary exposure is unvaccinated participants. Reference group for year of diagnosis is 2017. * *p* < 0.05, ** *p* < 0.01, *** *p* < 0.001.

**Table 3:** Multivariable Logistic Regression for Number of Varicella Doses and Reported Contact History.

Characteristic	Reported Contact with Varicella or Zoster OR (95% CI)	Reported Contact with Varicella OR (95% CI)	Reported Contact with Zoster OR (95% CI)
**1 dose (vs. 0 doses)**	1.34 ** (1.13, 1.60)	1.36 ** (1.12, 1.66)	1.17 (0.91, 1.52)
**2 doses (vs. 0 doses)**	1.35 ** (1.10, 1.65)	1.35 * (1.06, 1.71)	1.20 (0.90, 1.60)
**Sex, male (vs. female)**	1.04 (0.90, 1.19)	1.15 (0.98, 1.35)	0.86 (0.70, 1.04)
**Age, per 1-year increase**	1.01 (0.99, 1.02)	1.02 *** (1.01, 1.04)	0.97 *** (0.96, 0.99)
**Observations (N)**	3696	3696	3696

*Note.* OR = Odds Ratio; CI = Confidence Interval. Reference group for the primary exposure is 0 doses (unvaccinated). All models were adjusted for year of diagnosis (categorical variable, individual year estimates not shown for brevity). * *p* < 0.05, ** *p* < 0.01, *** *p* < 0.001.

**Table 4:** Multivariable Logistic Regression for Time Since First Varicella Dose and Reported Contact History.

Characteristic	Reported Contact with Varicella or Zoster OR (95% CI)	Reported Contact with Varicella OR (95% CI)	Reported Contact with Zoster OR (95% CI)
**1–3 years (vs. 43 days–1 year)**	2.21 (0.86, 5.65)	1.50 (0.50, 4.51)	2.86 (0.64, 12.80)
**3–5 years (vs. 43 days–1 year)**	2.43 (0.95, 6.24)	1.73 (0.58, 5.22)	2.91 (0.65, 13.12)
**5–10 years (vs. 43 days–1 year)**	2.91 * (1.15, 7.40)	2.07 (0.70, 6.15)	3.13 (0.70, 14.02)
**≥10 years (vs. 43 days–1 year)**	3.17 * (1.20, 8.41)	2.81 (0.91, 8.70)	2.30 (0.48, 11.01)
**Sex, male (vs. female)**	1.06 (0.90, 1.26)	1.13 (0.93, 1.38)	0.94 (0.75, 1.19)
**Age, per 1-year increase**	0.98 (0.95, 1.01)	1.00 (0.96, 1.03)	0.97 (0.93, 1.02)
**Observations (N)**	2470	2470	2470

*Note.* OR = Odds Ratio; CI = Confidence Interval. Reference group for the primary exposure is participants with time since first dose of 43 days–1 year. All models were adjusted for year of diagnosis (categorical variable, individual year estimates not shown for brevity). Analysis was based on a consistent sample size (*N* = 2470). * *p* < 0.05.

**Table 5:** Multivariable Logistic Regression for Time Since Last Varicella Dose and Reported Contact History.

Characteristic	Reported Contact with Varicella or Zoster OR (95% CI)	Reported Contact with Varicella OR (95% CI)	Reported Contact with Zoster OR (95% CI)
**1–3 years (vs. 43 days–1 year)**	2.11 (0.97, 4.59)	2.02 (0.69, 5.91)	1.80 (0.66, 4.89)
**3–5 years (vs. 43 days–1 year)**	2.38 * (1.09, 5.18)	2.36 (0.81, 6.91)	1.87 (0.68, 5.11)
**5–10 years (vs. 43 days–1 year)**	2.94 ** (1.36, 6.36)	3.19 * (1.11, 9.22)	1.85 (0.68, 5.08)
**≥10 years (vs. 43 days–1 year)**	3.17 ** (1.39, 7.22)	4.15 * (1.37, 12.54)	1.37 (0.46, 4.13)
**Sex, male (vs. female)**	1.07 (0.90, 1.26)	1.14 (0.94, 1.38)	0.94 (0.74, 1.19)
**Age, per 1-year increase**	0.98 (0.95, 1.01)	0.99 (0.96, 1.02)	0.97 (0.93, 1.02)
**Observations (N)**	2470	2470	2470

*Note.* OR = Odds Ratio; CI = Confidence Interval. Reference group for the primary exposure is participants with time since last dose of 43 days–1 year. All models were adjusted for year of diagnosis (categorical variable, individual year estimates not shown for brevity). Analysis was based on a consistent sample size (*N* = 2470). * *p* < 0.05, ** *p* < 0.01.

## Data Availability

The analytical data were retrieved from two official platforms operated by the Chaoyang District Center for Dis-ease Control and Prevention in Beijing: the Immunization Programme Database and the Infectious Disease Surveil-lance System. In accordance with local health information management policies, the original underlying data can-not be disclosed or shared externally.

## Supplementary Materials

The following supporting information can be downloaded at: https://www.mdpi.com/article/10.3390/vaccines14070617/s1. Table S1. Sensitivity Analysis (Unreported Vaccination Status Coded as Unvaccinated); Table S2. Epidemiological Case Investigation Form for Suspected Varicella/Herpes Zoster Cases.
