# Supplementary material for: Association Between Varicella Vaccination Status and Self-Reported Contact History Among Confirmed Varicella Cases in Chaoyang District, Beijing, 2017–2025
Source: Vaccines (Basel). 2026 Jul 14;14(7):617. doi: 10.3390/vaccines14070617 (PMC13417107; doi:10.3390/vaccines14070617)
Supplement: Supplementary file 1 [file vaccines-14-00617-s001.zip › vaccines-4402715-supplementary.pdf]

Table S1. Sensitivity Analysis (Unreported Vaccination Status Coded as Unvaccinated).

Characteristic	Reported Contact with Varicella OR (95% CI)
**Vaccinated (vs. Unvaccinated, including unreported)**	1.52*** (1.28, 1.79)
**1 dose (vs. 0 doses, including unreported)**	1.53*** (1.28, 1.82)
**2 doses (vs. 0 doses, including unreported)**	1.49*** (1.20, 1.85)
**Adjustments (Age, Sex, Year)**	Yes
**Observations (N)**	4,441

Note. OR = Odds Ratio; CI = Confidence Interval. In this sensitivity analysis, participants with unreported varicella vaccination status were coded as unvaccinated. All models were adjusted for age (years), sex (male vs. female), and year of diagnosis. Results are consistent with the primary analysis, indicating the robustness of our findings. *** $p < 0.001$.

Table S2. Epidemiological Case Investigation Form for Suspected Varicella/Herpes Zoster Cases

District _________, Township (Sub-district) _________, Village (Residential Committee) _________,

National District Code* ________________________ □□□□□□

Township (Sub-district) Code* ________________________ □□

Year* ________________________ □□□□

Case Serial No.* ________________________ □□□□

Case ID (Auto-generated from the above four items)

Case Type: ① Varicella ② Herpes Zoster □

I. Case Investigation Information

Date of Report*: ____ Year ____ Month ____ Day □□/□□/□□

Reporting Institution*: ________________

1. Date of Investigation*: ____ Year ____ Month ____ Day □□/□□/□□

Investigating Institution*: ________________

2. Investigator(s)*: ________________
3. Patient Name*: ________________
4. Notifiable Infectious Disease Card No. (Fill in for varicella cases only): □□□□□□□□□—□□□□—□□□□□
5. Date of Birth*: ____ Year ____ Month ____ Day □□/□□/□□

Or Age (Fill only if date of birth unavailable): ____ years (Convert months to years, retain 2 decimal places) □□.□□

6. Gender*: ① Male ② Female □
7. Occupation*:

(1) Kindergarten Child (2) Non-institutional Child (3) Primary School Student (4) Middle School Student

(5) University Student (6) Teacher (7) Medical Staff (8) Cadre & Clerk (9) Housewife/Unemployed

(10) Commercial Service Worker (11) Factory Worker (12) Catering & Food Industry Worker

(13) Public Place Attendant (14) Retiree (15) Farmer (16) Nanny & Babysitter

(17) Migrant Worker (18) Herdsman (19) Fisherman (20) Seaman & Long-distance Driver

(21) Others □

If student:

Full School Name: ________________ Class: ________________

School Jurisdiction: District/County: ________ Township (Sub-district): ________

Suspension from School: ① Yes ② No □

Date of School Suspension: ____ Year ____ Month ____ Day □□/□□/□□

Date of Resumption (Fill during second follow-up): ____ Year ____ Month ____ Day □□/□□/□□

If non-student: Work Unit: ___________________________

9. Residential Address: ___________________________

10. Lost to Follow-up*: ① Yes ② No □

11. Household Registration*: ① Local Resident ② Other Province: ________ ⑨ Foreign National: _______ □

12. Interval from Arrival to Onset*: ① Within 3 weeks ② Over 3 weeks □

13. Guardian Names: Father: ________ Mother: ___________

14. Contact Phone: _______________

II. Clinical Manifestations

1. Fever Present*: ① Yes ② No □

2. Date of Fever Onset: ____ Year ____ Month ____ Day □□/□□/□□

Maximum Body Temperature: ____ ℃ □□.□

Duration of Fever (Fill during second follow-up): ____ days □□

3. Date of Rash Onset*: ____ Year ____ Month ____ Day □□/□□/□□

4. Rash Morphology*:

① Papule ② Vesicle ③ Papule + Vesicle ④ Macule

⑤ Papulovesicle (Macular base with tiny vesicles on top)

⑥ Macule + Papule only ⑨ Mixed maculopapules and vesicles □

5. Rash Severity*:

1 Mild (<50 scattered lesions): ____ lesions

2 Moderate (50–249 lesions)

3 Moderately Severe (250–499 lesions)

4 Severe (Extensive palpable rashes with no normal skin between lesions) □

6. Rash Distribution (Record order of appearance):

Face & Head____ Chest___ Back__ Abdomen__ Upper Limbs__ Lower Limbs__ Hands & Feet__ Other__

7. Oral Mucosa Involvement: ① Yes ② No ⑨ Unknown □

8. Pruritus Associated with Rash: ① Yes ② No ⑨ Unknown □

9. Other Symptoms (Multiple selections allowed):

① None□ ② Sore Throat□ ③ Cough□ ④ Vomiting□

⑤ Anorexia□ ⑥ Myalgia□ ⑦ Fatigue□ ⑧ Others____□

10. Complications*

10.1 Headache: ① Yes ② No ⑨ Unknown □

10.2 Fatigue: ① Yes ② No ⑨ Unknown □

10.3 Anorexia: ① Yes ② No ⑨ Unknown □

10.4 Cough: ① Yes ② No ⑨ Unknown □

10.5 Dehydration: ① Yes ② No ⑨ Unknown □

10.6 Pneumonia: ① Yes ② No ⑨ Unknown □

10.7 Encephalitis: ① Yes ② No ⑨ Unknown □

10.8 Skin Secondary Infection: ① Yes ② No ⑨ Unknown □

10.9 Cerebellar Ataxia: ① Yes ② No ⑨ Unknown □

10.10 Other Secondary Infections: ① Yes ② No ⑨ Unknown □

10.11 Thrombocytopenia: ① Yes ② No ⑨ Unknown □

10.12 Other Complications: ① Yes ② No ⑨ Unknown □

11. Antiviral treatment with Acyclovir, Valacyclovir, etc. during this illness: ① Yes ② No □

If Yes, complete below:

Drug 1 Name: ____________

Treatment Start Date: ____ Year ____ Month ____ Day □□□□/□□/□□

Treatment End Date: ____ Year ____ Month ____ Day □□□□/□□/□□

12. Hospitalization*: ① Yes ② No ⑨ Unknown □

If hospitalized:

Admission Date: ____ Year ____ Month ____ Day □□□□/□□/□□

Discharge Date: ____ Year ____ Month ____ Day □□□□/□□/□□

Primary Discharge Diagnosis: ____________________________

Co-existing Diagnoses (Multiple allowed):

① Varicella□ ② Post-varicella Encephalitis□ ③ Varicella Pneumonia□ ④ None□

13. Prior History of Varicella*: ① Yes, Year of illness: ________ ② No □

14. Underlying Diseases (Multiple allowed)*:

① None□ ② Leukemia / Other Malignancy□ ③ Severe T-cell Immunodeficiency□

④ Hematopoietic Stem Cell / Solid Organ Transplant Recipient□ ⑤ Chemotherapy□ ⑥ HIV Infection□

15. Long-term Corticosteroid or Immunosuppressant Medication*: ① Yes ② No □

III. Epidemiological History

1. Exposure to confirmed cases within 3 weeks before onset*:

① Lab/clinically confirmed varicella case ② Suspected varicella case ③ Herpes zoster patient ⑨ Unknown □

If Yes: Days of exposure before rash onset: ____ days □□

Exposure Venue: ① Hospital ② School ③ Home ④ Community ⑤ Other: ______ □

Exposure Type (Multiple allowed):

① Group activities (play)□ ② Shared meals□ ③ Commute to/from school together□

④ Neighboring residence□ ⑤ Other (specify): ____□

2. Hospital visit 7–21 days before rash onset*: ① Yes ② No □

Hospital Names & Dates:

3. ____________ (Date: ___ Month ____ Day)

4. ____________ (Date: ___ Month ____ Day)

5. ____________ (Date: ___ Month ____ Day)

IV. Vaccination History

1. Source of Vaccination Record*:

① Vaccination Book ② Vaccination Card (Information System) ③ Guardian Recall ④ Other □

If Option ②: Child ID in Immunization Information System: □□□□□□□□□□□□□□□□□□□□

2. Received Varicella Vaccine*: ① Yes ② No ⑨ Unknown □

If Yes:

a. Number of Doses: ① 1 dose ② 2 doses ③ 3 doses or more □

b. Date of 1st Dose: ____ Year ____ Month ____ Day

OR Interval from vaccination to onset:

① ≤42 days ② 43 days to <1 year ③ 1 year to <3 years

④ 3 years to <5 years ⑤ 5 years to <10 years ⑥ ≥10 years ⑨ Unknown □

c. Venue of 1st Dose:

① Local, District: _____ Community: ______ ② Other Province: ______ ③ Other: _____ □

d. Vaccine Brand for 1st Dose: ① Imported ② Domestic, Manufacturer: ______ ⑨ Unknown □

e. Date of 2nd Dose: ____ Year ____ Month ____ Day

OR Interval from vaccination to onset:

① ≤42 days ② 43 days to <1 year ③ 1 year to <3 years

④ 3 years to <5 years ⑤ 5 years to <10 years ⑥ ≥10 years ⑨ Unknown □

f. Venue of 2nd Dose:

① Local, District: _____ Community: ______ ② Other Province: ______ ③ Other: _____ □

g. Vaccine Brand for 2nd Dose: ① Imported ② Domestic, Manufacturer: ______ ⑨ Unknown □

3. Has District CDC verified complete 2-dose vaccination history? ① Yes ② No □

CDC Verification Date (Auto-generated): ____ Year ____ Month ____ Day

Investigator: __________________

V. Outbreak Classification

① Sporadic ② Outbreak ③ Public Health Emergency

(School outbreaks are defined until 21 days after the last case's rash onset) □

Outbreak/Emergency Serial No.

(District Code + Year + 2-digit serial): □□□□□□—□□□□—□□
